# La fibroscopie digestive haute chez 2795 patients au centre hospitalier universitaire-campus de Lomé: les particularités selon le sexe

**DOI:** 10.11604/pamj.2014.19.262.4512

**Published:** 2014-11-10

**Authors:** Laté Mawuli Lawson-Ananissoh, Oumboma Bouglouga, Aklesso Bagny, Laconi Kaaga, Datouda Redah

**Affiliations:** 1 Service d'Hépato-gastro-entérologie du CHU Campus de Lomé, Togo

**Keywords:** Fibroscopie digestive haute, indications, particularités, sexe, Togo, Upper gastrointestinal endoscopy in 2795 patients at the university campus hospital of Lomé: peculiarities according to sex, indications, Upper gastrointestinal endoscopy in 2795 patients at the university campus hospital of Lomé: peculiarities according to sex, sex, Togo

## Abstract

**Introduction:**

Notre étude consistera à rapporter les indications et les lésions objectivées à la fibroscopie digestive haute et relever les particularités selon le sexe.

**Méthodes:**

Étude rétrospective, descriptive sur des résultats de compte-rendu de la fibroscopie digestive haute menée en unité d'endoscopie digestive du service d'hépato-gastro-entérologie du CHU Campus de Lomé du 15 Mai 2009 au 31 Décembre 2013.

**Résultats:**

La fibroscopie digestive haute a été réalisée chez 2795 patients dont 1188 hommes et 1607 femmes. L’âge moyen était de 40,65 ans (Extrêmes: 5 et 93 ans). La fibroscopie digestive haute était normale chez les femmes que chez les hommes avec une différence statistiquement significative (p = 0,000). Les principales indications étaient: les épigastralgies chez les femmes (p = 0,000); les hémorragies digestives hautes (p = 0,000) et l'hypertension portale (p = 0,000) chez les hommes; 3485 lésions pathologiques ont été observées. La pathologie inflammatoire prédominait (56,3%), la pathologie ulcéreuse (13,89%), la pathologie tumorale (2,01%). Les varices et la candidose œsophagiennes étaient significativement notées chez les hommes. Les ulcérations gastriques (p = 0,000), le reflux biliaire duodéno-gastrique (p = 0,017) étaient plus retrouvés chez les femmes et la gastropathie hypertensive beaucoup plus chez les hommes (p = 0,000). Que les lésions duodénales soient inflammatoires ou ulcéreuses associées ou non à une sténose bulbaire, elles étaient plus fréquentes chez les hommes.

**Conclusion:**

De manière générale, il y avait une prédominance des lésions inflammatoires chez les femmes, les lésions tumorales et ulcéreuses chez les hommes

## Introduction

La fibroscopie digestive haute est une méthode d'exploration visuelle du tractus digestif haut (œsophage, estomac, duodénum). Grace à cet examen, la prise en charge des hémorragies digestives hautes et les hépatopathies chroniques est améliorée. Bien que cet examen performant soit de pratique courante dans les pays occidentaux, il n'est malheureusement pas encore généralisé en milieu tropical en général et au Togo en particulier. Le Togo avec environ six millions d'habitants ne dispose que de trois gastro-entérologues. La pratique de la fibroscopie digestive haute au Togo est concentrée à Lomé la capitale. En 2001, une étude menée par Redah et al avait montré l'apport de l'endoscopie dans le diagnostic de l’œsophagite à Candida albicans [1]. Il nous a donc semblé opportun de mener cette étude en unité d'endoscopie digestive du service d'hépato-gastro-entérologie du Centre Hospitalier Universitaire (CHU) Campus de Lomé afin de faire le point sur la pratique de l'endoscopie digestive haute dans l'exploration des pathologies digestives hautes au Togo ces cinq dernières années. Il s'agissait spécifiquement de déterminer les principales caractéristiques sociodémographiques des patients ayant bénéficié de la fibroscopie digestive haute, de relever les indications et les lésions œsogastroduodénales retrouvées et d'expliquer les particularités notées selon le sexe.

## Méthodes

Il s'agit d'une étude rétrospective, descriptive sur des résultats de compte-rendu de fibroscopie digestive haute menée en unité d'endoscopie digestive du service d'hépato-gastro-entérologie du CHU Campus de Lomé du 15 Mai 2009 au 31 Décembre 2013. Les examens endoscopiques ont été effectués par un gastroentérologue confirmé assisté d´une aide. L'endoscopie a été réalisée sans sédation, avec une anesthésie oro-pharyngée par gargarisme de 2 ml de Xylocaïne gel associée ou non à une prémédication par du métopimazine en intraveineuse directe. Le matériel utilisé était un fibroscope “FUJINON-EG-/250WRVIDEO” à vision axiale et à la lumière froide. Les patients se présentaient le matin à jeun depuis la veille de l'examen sans autre préparation spéciale. Des biopsies per-endoscopiques ont été réalisées chaque fois que cela a été possible. Les fragments de biopsie étaient fixés au formol à 10% et adressés au laboratoire d'anatomie pathologique du CHU- Sylvanus Olympio de Lomé. La désinfection du matériel après utilisation était réalisée selon le protocole de désinfection manuelle préconisé par la Société Française d'Endoscopie Digestive (SFED) en plaçant l'endoscope et ses accessoires dans une solution d'Hexanios^®^ avec brossage des accessoires et la stérilisation était réalisée dans une solution de Steranios^®^ 2% (glutaraldéhyde).

Etaient inclus les patients des deux sexes reçus en unité d'endoscopie digestive, ayant bénéficié de l'endoscopie digestive haute et dont le compte rendu comportait: -les données socio- démographiques: nom et prénoms, âge, sexe, provenance; -l'indication ou les indications de la fibroscopie digestive haute; -le consentement verbal du patient; -le détail de l'examen: description faite par niveau; -œsophage, estomac, duodénum; -la conclusion.

La saisie des données a été faite avec EXCEL 2013; l'analyse statistique a été faite avec le logiciel STATA version 2012 et le test de Khi 2 a été utilisé pour comparer nos résultats qui étaient significatifs pour une probabilité p < 0,05.

## Résultats

Au cours de la période d’étude, la fibroscopie digestive haute a été réalisée chez 2795 patients dont 1188 hommes et 1607 femmes. L’âge moyen était de 40,65 ans avec des extrêmes de 5 et 93 ans. La tranche d’âge la plus représentée était celle de 25 à 44 ans avec 1336 patients (47,8%). La majorité des patients soit 2143 patients (76,67%) avaient bénéficié de la fibroscopie digestive haute à titre externe; 556 patients (19,89%) étaient hospitalisés dans le service d'hépato-gastro-entérologie et 59 patients (2,11%) provenaient du service de médecine interne. Les principales indications de la fibroscopie digestive haute étaient: les épigastralgies (60,68%), les hémorragies digestives hautes (9,23%), la douleur abdominale diffuse (7,19%) et l'hypertension portale (5,9%). Les femmes se plaignaient plus pour les douleurs abdominales (épigastriques, diffuses ou rétrosternales) alors que les hémorragies digestives hautes et l'hypertension portale étaient plus rapportées par les hommes. Il y avait une différence statistiquement significative entre les hommes et les femmes par rapport aux épigastralgies (p = 0,000), les hémorragies digestives hautes (p = 0,000) et l'hypertension portale (p = 0,000). Les principales indications de la fibroscopie digestive haute en fonction du sexe sont représentées dans le Tableau 1.

**Tableau 1:** Principales indications de la fibroscopie digestive haute selon le sexe

	Femmes	Hommes	Total	%	Khi2	p
**Epigastralgie**	1107	589	1696	60,68	106,71	0,000
**Hémorragies digestives hautes**	94	164	258	9,23	51,59	0,000
**Douleur abdominale diffuse**	107	94	201	7,19	1,6	0,2
**Hypertension portale**	51	114	165	5,9	50,71	0,000
**Vomissements**	68	46	114	4,08	0,22	0,63
**Douleur rétrosternale**	60	31	91	3,26	2,74	0,098
**Dysphagie**	29	30	59	2,11	1,71	0,19
**RGO**	33	19	52	1,86	0,77	0,38
**Précordialgie**	20	16	36	1,29	0,05	0,81
**Anémie**	16	14	30	1,07	0,21	0,64
**Altération de l’état général**	10	15	25	0,89	3,15	0,075
**Rectorragie**	9	8	17	0,61	0,14	0,39
**Hocquet**	4	13	17	0,61	8,07	0,004
**Ingestion caustique**	7	9	16	0,57	1,24	0,26
**Contrôle**	11	5	16	0,57	0,83	0,36
**Masse abdominale**	4	9	13	0,47	3,81	0,051
**Tentative de suicide**	5	6	11	0,39	0,65	0,41
**Métastases hépatiques**	3	4	7	0,25	0,61	0,43
**Maladie de Biermer**	5	0	5	0,18	3,7	0,054
**Corps étranger dans la gorge**	4	1	5	0,18	1,03	0,3

Au cours de la fibroscopie digestive haute, 3485 lésions pathologiques ont été observées. La pathologie inflammatoire (‘sophagite, gastropathie, bulbo-duodénite) prédominait avec 56,3%, les pathologies ulcéreuse, tumorale et les lésions diverses regroupées représentaient respectivement 13,89%, 2,01% et 27,8%. (Tableau 2) Les lésions endoscopiques étaient réparties comme suit: L'estomac avec 2425 lésions (69,58%); L’œsophage avec 308 lésions (8,84%); Le duodénum avec 751 lésions (21,55%); Une lésion située au niveau de la bouche de Killian (0,03%).

**Tableau 2:** Type pathologique selon le sexe

	Femme		Homme		Total	
	n	%	n	%	n	%
Inflammatoire	1061	54,08	901	45,92	1962	56,3
Ulcéreuse	219	45,25	265	54,75	484	13,89
Tumorale	33	47,14	37	52,86	70	2,01
Diverses	526	54,39	443	45,71	969	27,8
Total	1839	52,77	1646	47,23	3485	100

L'endoscopie digestive haute était plus souvent décrite comme normale chez les femmes que chez les hommes avec une différence statistiquement significative (p = 0,000) comme le montre le Tableau 3. Une lésion tumorale a été objectivée au niveau de la bouche de Killian chez une femme de 65 ans Tableau 3). Au niveau de l’œsophage, la fibroscopie digestive haute est plus souvent normale chez la femme que chez les hommes (p = 0,000); on notait une différence significative entre les hommes et les femmes en ce qui concerne les varices œsophagiennes et la candidose; toutes deux étaient plus retrouvées chez les hommes que chez les femmes (Tableau 3).

**Tableau 3:** Comparaison des lésions objectivées à la fibroscopie digestive haute selon le sexe

	Femmes	Hommes	Total	Khi 2	p	Significativité
Bouche de Killian	1	0	1	0,73	0,39	NS
**Œsophage**						
Normal	1486	1000	2486	47,79	0,000	S
Hernie hiatale	19	15	34	0,03	0,84	NS
Œsophagite	23	23	46	1,07	0,3	NS
Candidose	13	25	38	8,54	0,003	S
Varices œsophagiennes	38	101	139	54,43	0,000	S
Tumeur	10	12	22	1,31	0,25	NS
Ulcère	2	5	7	2,4	0,12	NS
Mallory-Weiss	5	6	11	0,65	0,41	NS
Béance cardiale	7	1	8	2,95	0,08	NS
Nécrose caustique	1	0	1	0,73	0,39	NS
Mégaoesophage	1	1	2	0,04	0,83	NS
**Estomac**						
Normal	545	396	941	0,1	0,74	NS
Congestion	319	253	572	0,87	0,34	NS
Erosion	332	242	574	0,03	0,85	NS
Ulcération	117	60	177	5,72	0,017	S
Ulcère	100	90	190	1,97	0,16	NS
Tumeur	18	21	39	2,08	0,149	NS
RBDG	353	206	559	9,13	0,000	S
Sténose antro-pylorique	8	13	21	3,25	0,07	NS
Gastropathie hypertensive	52	99	151	34,72	0,000	S
Hypersécrétion gastrique	57	44	101	0,04	0,82	NS
Stase alimentaire	5	5	10	0,23	0,63	NS
Atrophie muqueuse	12	12	24	0,55	0,45	NS
Varices gastriques	2	3	5	0,62	0,42	NS
Nécrose caustique	1	1	2	0,04	0,83	NS
**Duodénum**						
Normal	1343	879	2222	38,47	0,000	S
Bulbite	142	146	288	8,81	0,003	S
Duodénite	76	78	154	4,42	0,035	S
Ulcère du bulbe	109	151	260	28,44	0,000	S
ulcère du 2è duodénum	8	19	27	8,66	0,003	S
Sténose bulbaire	4	10	14	4,81	0,028	S
Tumeur	4	4	8	0,18	0,66	NS
						
**Endoscopie normale**	417	206	623	29,22	0,000	S

RBDG: Reflux biliaire duodéno-gastrique S: Significatif NS: Non significatif

Au niveau de l'estomac, les ulcérations (p = 0,017) et le reflux biliaire duodéno-gastrique (p = 0,000) étaient significativement plus retrouvés chez les femmes que chez les hommes; les ulcérations gastriques étaient deux fois plus fréquentes chez les femmes. La gastropathie hypertensive était notée beaucoup plus chez les hommes (p = 0,000) (Tableau 3). Au niveau du duodénum, les résultats normaux étaient plus fréquents chez les femmes que chez les hommes. Que les lésions soient inflammatoires ou ulcéreuses associées ou non à une sténose bulbaire, elles étaient plus retrouvées chez les hommes que chez les femmes et la différence était statistiquement significative (Tableau 3).

## Discussion

Reçoit en unité d'endoscopie des patients hospitalisés dans ledit centre hospitalier et des patients provenant de diverses régions du pays. Ce travail réalisé en unité endoscopique nous montre que la pathologie digestive haute au Togo est dominée par les pathologies inflammatoires puis viennent les pathologies ulcéreuses. Nous avons retrouvé dans notre population d’étude une prédominance féminine tout comme dans la série nigériane [2]; ceci est contraire à des séries africaines [3–6] et asiatiques [7] qui avaient retrouvé une prédominance masculine. Nos patients étaient jeunes avec une moyenne d’âge de 40,65 ans et la tranche d’âge la plus représentée était comprise entre 25 et 44 ans. Ce résultat est proche des données de la plupart des études africaines [3, 5, 6] et asiatiques [8, 9]. Ceci pourrait s'expliquer par la structure de la population des pays en développement avec un fort pourcentage des sujets jeunes dans la population. De plus, les sujets jeunes consulteraient beaucoup plus que les sujets âgés qui sont plus orientés vers la médecine traditionnelle. La fibroscopie digestive haute était normale chez 623 patients (22,29%), ce taux est supérieur à celui noté par Ismaila (15,6%) [10] mais inférieur à celui noté par Taye (28%) [5], Aduful (41,1%) [4] et Shah (78,5%) [9]; de plus l'endoscopie digestive haute était significativement décrite comme normale chez les femmes que chez les hommes dans notre série. Cette prédominance de résultats normaux chez les femmes a été également objectivée dans les études béninoise, pakistanaise et indienne [3, 8, 9]. Ces divergences observées pourraient être dues à une indication plus large de la fibroscopie digestive haute ou aux manifestations des parasitoses digestives, les troubles fonctionnels intestinaux et les mauvaises habitudes alimentaires. La fréquence habituellement élevée des troubles psychosomatiques avec des troubles fonctionnels chez les femmes pourrait également expliquer la prédominance féminine des résultats normaux au cours de la fibroscopie digestive haute.

Les épigastralgies ont constitué le principal motif de réalisation de la fibroscopie digestive haute dans notre étude tout comme dans les autres séries [3, 4, 6] mais à un taux largement supérieur à celui de ces séries. Contrairement à certaines séries [2, 5, 7–9, 11, 12], c'est la dyspepsie qui était la principale indication de la fibroscopie digestive haute. Dans notre étude, la pathologie inflammatoire prédominait (56,3%) comme il a été constaté dans les séries d'Alandry (49,5%) [6] et Ismaila (39,3%) [10]. Cette fréquence élevée pourrait s'expliquer par la fréquence élevée du reflux biliaire gastroduodénal dans notre série d'une part et d'autre part par l'automédication aux produits gastro-toxiques et les mauvaises habitudes alimentaires. Nous avons noté de façon globale une prédominance des lésions inflammatoires chez les femmes et une prédominance des lésions ulcéreuses et tumorales chez les hommes. Ceci a été également constaté dans les séries africaines et asiatiques [3, 9, 13]. Quand bien même nous sommes dans un pays en voie de développement où il y a une forte prévalence de l'infection à Hélicobacter pylori [14], notre étude rétrospective, ne nous a pas permis d'affirmer la part de l'Hélicobacter pylori dans la genèse de ces lésions tant chez les femmes que chez les hommes au Togo, mais aussi elle ne nous permet pas d'affirmer sa prédominance chez les femmes. Des études devront donc être menées pour renseigner sur la part de l'Hélicobacter pylori dans la survenue de ces lésions au sein de la population togolaise. Toutefois l'automédication à base des anti-inflammatoires non stéroïdiens, leur usage dans la gestion des dysménorrhées chez la femme et pour d'autres indications pourraient expliquer la survenue des lésions inflammatoires chez celle-ci. Notre étude en outre ne nous a pas permis d’évaluer la part de la prise d'alcool et du tabac dans la survenue des lésions objectivées à la fibroscopie digestive haute. Nous avons constaté au niveau de l’œsophage, qu'il existait une différence significative entre les hommes et les femmes en ce qui concerne les varices œsophagiennes et la candidose; toutes deux étaient plus retrouvées chez les hommes que chez les femmes; ce qui est conforme aux données de la littérature. Boghratian et al en Iran avaient noté que les varices œsophagiennes étaient retrouvées chez les hommes que chez les femmes par contre dans la série indienne, il a été noté seulement 2 cas de varices œsophagiennes et c’était chez des femmes [9]; la durée de cette étude indienne (02 ans) pourrait expliquer ce faible taux de varices œsophagiennes; les varices œsophagiennes sont des signes endoscopiques d'hypertension portale dont la principale cause est la cirrhose qui touche les hommes que les femmes [15].

Au niveau du duodénum, que les lésions soient inflammatoires ou ulcéreuses associées ou non à une sténose bulbaire, elles sont plus retrouvées chez les hommes que chez les femmes et la différence était statistiquement significative. Ce constat a été fait dans les séries africaines [3, 6]; Khurram et al au Pakistan avaient retrouvé également une prédominance de l'ulcère duodénal chez les hommes mais contrairement à notre série, les bulbo-duodénites étaient prédominantes chez les femmes [8].

## Conclusion

La fibroscopie digestive haute est un examen complémentaire important pour le gastro-entérologue dans l'exploration des pathologies digestives hautes; sa pratique au CHU Campus de Lomé a montré de manière générale la prédominance des lésions inflammatoires chez les femmes et les lésions tumorales et ulcéreuses chez les hommes. Différents facteurs comme la prise des médicaments gastro-toxiques, les mauvaises habitudes alimentaires pourraient expliquer la survenue de ces lésions; d'autres études pourront être initiées afin de préciser la place d'autres facteurs comme l'alcool, le tabac et l'Hélicobacter pylori dans l'explication des différences retrouvées entre les hommes et les femmes lors de la survenue des différentes lésions objectivées à la fibroscopie digestive haute.
